# Heparinized swine models for better surgical/endoscopic training

**DOI:** 10.1002/deo2.64

**Published:** 2021-10-25

**Authors:** Yuto Kubo, Kotaro Yamashita, Takuro Saito, Koji Tanaka, Tomoki Makino, Tsuyoshi Takahashi, Yukinori Kurokawa, Makoto Yamasaki, Hidetoshi Eguchi, Yuichiro Doki, Kiyokazu Nakajima

**Affiliations:** ^1^ Department of Next‐Generation Endoscopic Intervention (Project ENGINE) Center of Medical Innovation and Translational Research, Graduate School of Medicine, Osaka University Osaka Japan; ^2^ Department of Gastroenterological Surgery Graduate School of Medicine Osaka University Osaka Japan

**Keywords:** bleeding model, endoscopy, experiment, large animal

## Abstract

**Introduction:**

Animal experiments with large living animals are essential for the development of medical devices and the training of surgical procedures. Swine are frequently used in animal experiments due to their similar size and anatomy compared to humans. However, it is well known that swine has less local bleeding than humans. The aim of the study was to verify whether animal models with appropriate local bleeding capability could be established.

**Methods:**

The activated clotting time (ACT) was measured for eight swine (piglet, 35 kg) under general anesthesia. The flexible endoscope was advanced orally, and the gastric mucosa was intentionally traumatized to bleed by biopsy forceps, and the time until spontaneous hemostasis was obtained (mucosal bleeding time). Then, heparin (50 U/kg) was administered intravenously. After 10 min, the ACT was remeasured, and the gastric mucosa was again damaged to bleed by biopsy forceps. The mucosal bleeding time was remeasured. The above measurements were repeated until the ACT exceeded 200 s.

**Results:**

The median ACT values (seconds) were 83 (no heparin), 155 (50 U/kg heparin), and 204 (100 U/kg heparin), which were significantly increased. The median mucosal bleeding times (seconds) were 152 (no heparin), 283 (50 U/kg), and 423 (100 U/kg), which were significantly extended.

**Conclusion:**

A bleeding animal model for surgical and endoscopic training was successfully established by bolus heparin administration.

## INTRODUCTION

Animal experiments have contributed to the development of new medicines and therapies, and have been used to exemplify known medical procedures and identify specific biological phenomena.^1^ Investigation by using living animals in both research and education obviously enabled many important discoveries and contributed significantly to medicine.^2^ Recently, animal experiments have been considered essential for the development of medical devices and training for techniques such as surgical procedures.^3^

Swine are frequently used as laboratory animals because they are similar to humans physiologically and anatomically.^4^ However, there are challenges that bleeding in swine is difficult to induce,^5^, ^6^ because swine have a higher function of coagulation and aggregation than humans.^6^, ^7^, ^8^, ^9^ This has become one of the major limitations to perform appropriate experiments for the development of medical devices or the training for surgical and endoscopic procedures. Only sporadic and observational reports are limitedly available in the literature,^6^ and simple, reproducible, and stable swine bleeding models have not been fully established.

In clinical practice, heparin is mainly used as an acute treatment for thromboembolisms such as cerebral infarction and myocardial infarction. Previous studies showed that decreasing coagulation function improves symptoms and reduces the risk of recurrence. Hence, heparin is one of the essential drugs and the most widely used anticoagulant for thromboembolism worldwide.^10^, ^11^ Therefore, we hypothesized that appropriate administration of heparin to pigs may show local bleeding enhancement. The purpose of this study was to establish bleeding models using pigs that show local bleeding for surgical and endoscopic training by bolus heparin administration.

## MATERIALS AND METHODS

### Animals

The entire experimental protocol in the present study was approved by the institutional animal care and ethical review board. All procedures were conducted in a standard manner under general anesthesia in 3‐month‐old female swine (*n* = 8; average weight = 35 kg).^12^ Each swine received pretreatment and was humanely euthanized upon completion of the experiment.

### Procedure

**FIGURE 1:**
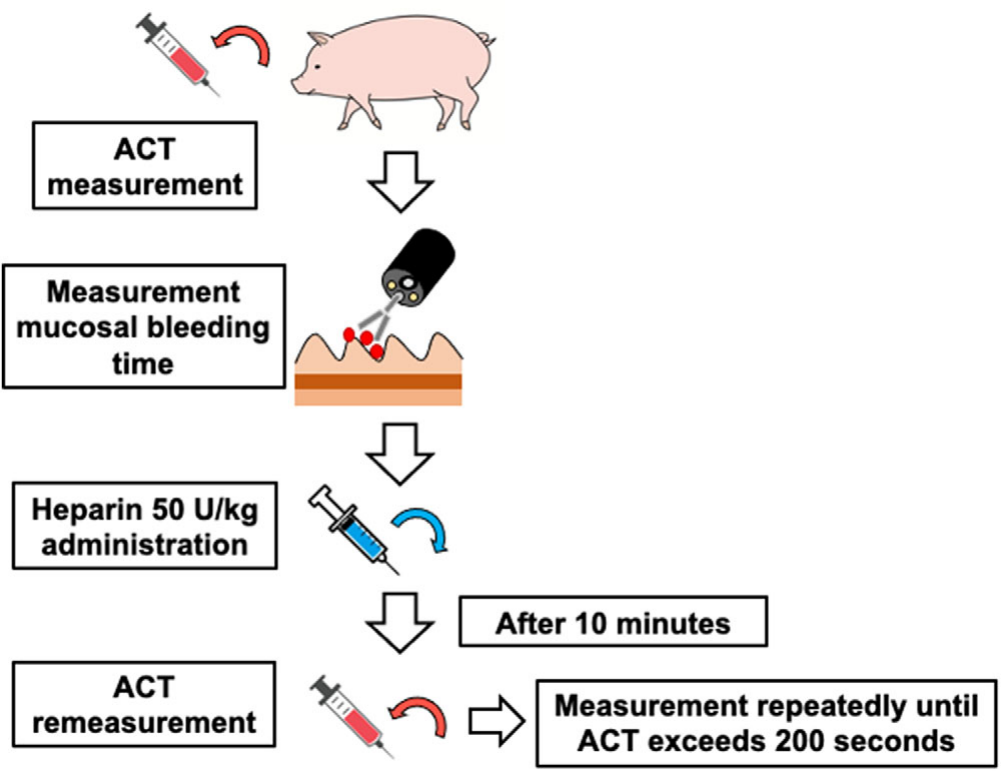
Experimental protocol

The activated clotting time (ACT) was measured in the swine under general anesthesia. A surgeon advanced an upper gastrointestinal endoscope (GIF‐260J; Olympus, Japan) orally and created exudative bleeding by using grasping forceps (FG‐47 L‐1; Olympus), which can grasp more deeply than standard biopsy forceps to make the mucosa easier to bleed, in the upper, middle, and lower anterior walls of the gastric mucosa, respectively. We defined a lack of exudative bleeding for 5 s as spontaneous hemostasis and washed the bleeding area with 0.9% saline (Otsuka Pharmaceutical Co. Ltd., Japan) under endoscopy. We measured the time until spontaneous hemostasis occurred (defined as mucosal bleeding time), and 50 U/kg unfractionated heparin (Heparin) was intravenously administered. After 10 min, the ACT and mucosal bleeding time were re‐examined, and 50 U/kg heparin was additionally re‐administered. The above measurements were repeated until the ACT exceeded 200 s (in press) while maintaining the systolic blood pressure at least 100 with a vasopressor (Figure 1).

### Statistical analysis

The Mann‐Whitney *U* test, chi‐square test, or Student's *t‐*test was used to compare the ACT and mucosal bleeding time. Continuous variables were expressed as the median (minimum value – maximum value) unless otherwise stated. A *p‐*value <0.05 was considered to indicate statistical significance. All analyses were performed using JMP 14 (SAS Institute Inc., Cary, NC, USA).

## RESULTS

Video S1 shows the endoscopic images that were compared for each heparin administration (no heparin administration vs. heparin total dose 50 U/kg vs. heparin total dose 100 U/kg). Video S1a demonstrates that exudative hemorrhage from mucous membranes in swine treated without heparin. Exudative bleeding was greater in swine treated with a total heparin dose of 50 U/kg than in those treated with a heparin dose of 0 U/kg (Video S1b). Moreover, Video S1c shows that blood traveled from the anterior wall to the posterior wall, resulting in more bleeding in swine treated with a total heparin dose of 100 U/kg than in those treated with a total heparin dose of 50 U/kg.

**FIGURE 2:**
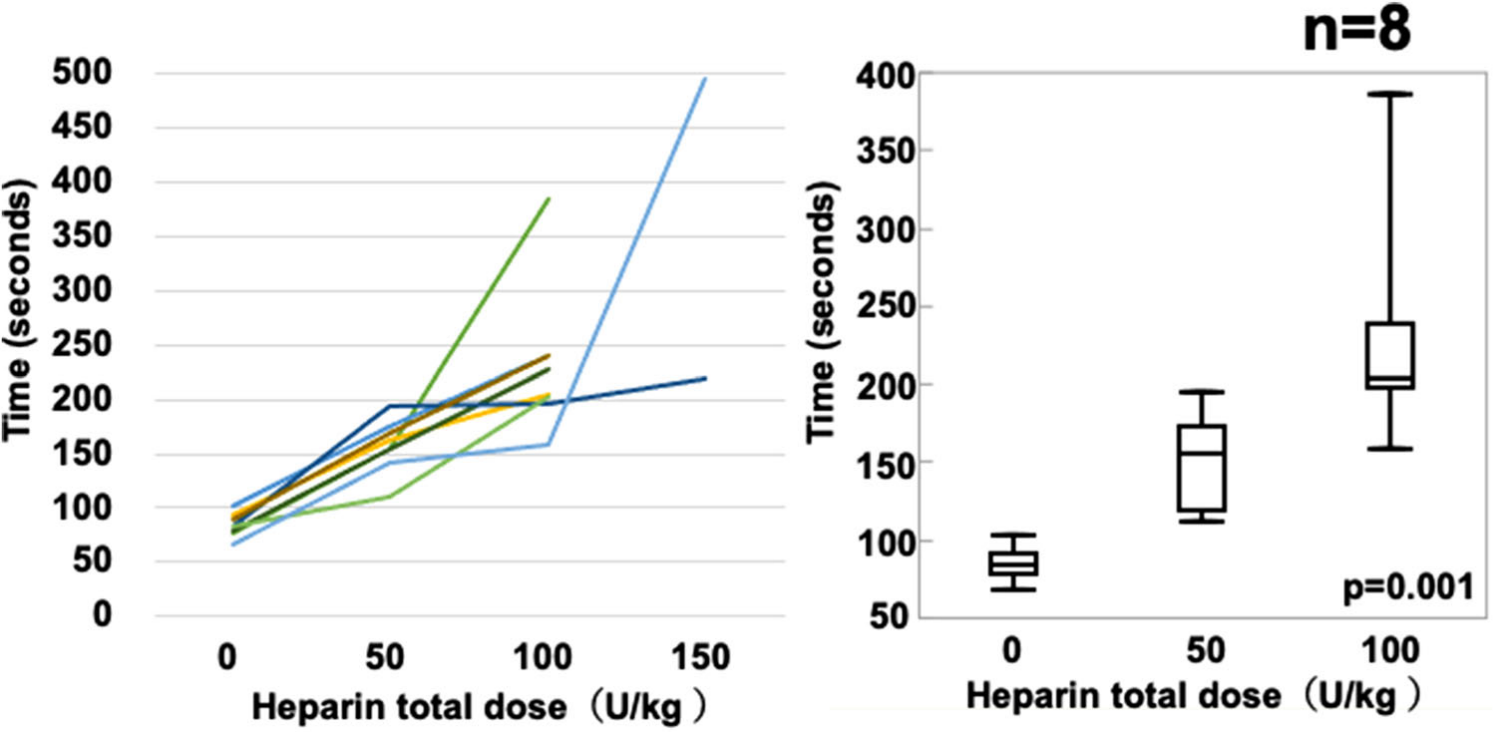
Changes in activated clotting time (ACT) for each heparin administration

Figure 2 summarizes the changes in the ACT (seconds) for each heparin administration. The median ACT was significantly increased by heparin administration (median ACT for no heparin vs. 50 U/kg vs. 100 U/kg: 83 vs. 155 vs. 204, respectively, *p* = 0.001)

**FIGURE 3:**
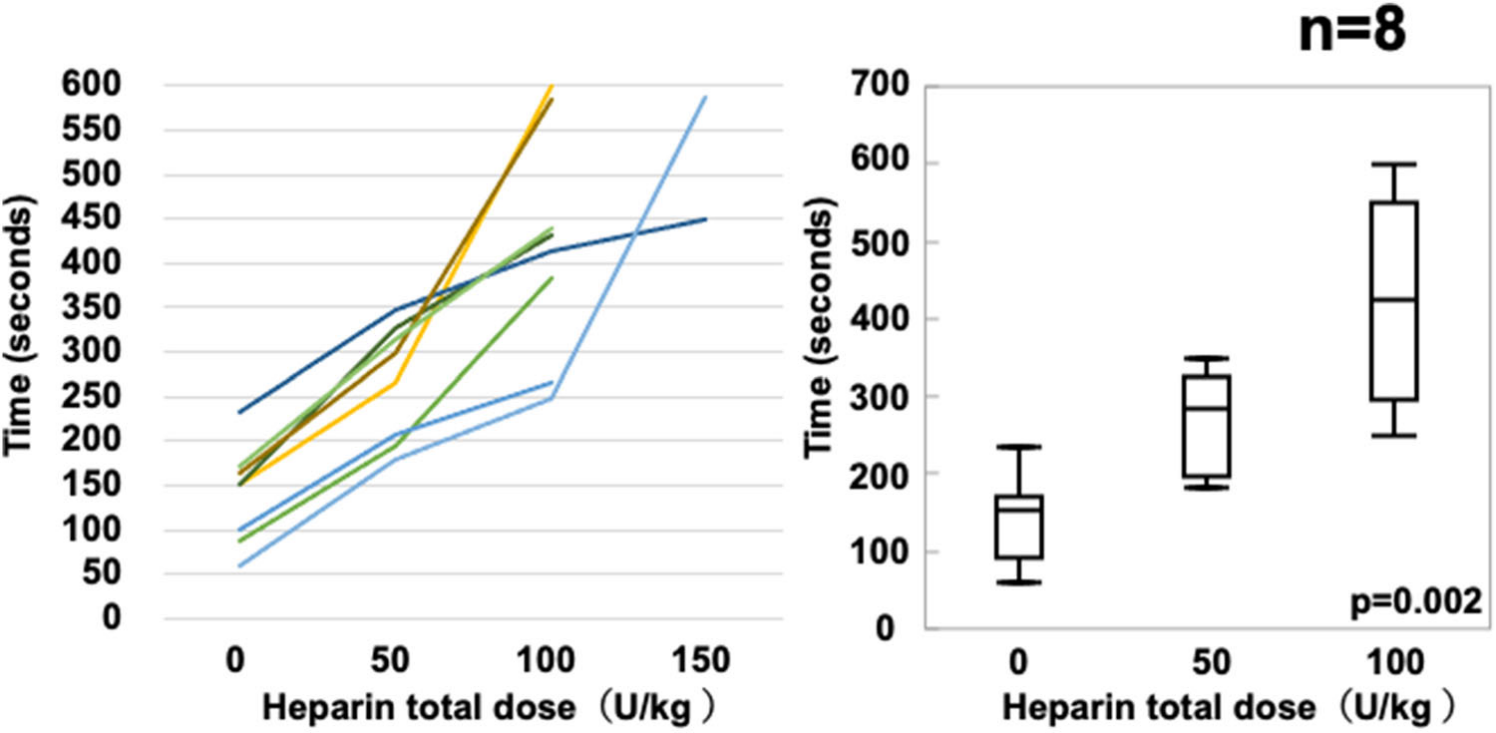
Changes in gastric mucosal bleeding time for each heparin administration

**FIGURE 4:**
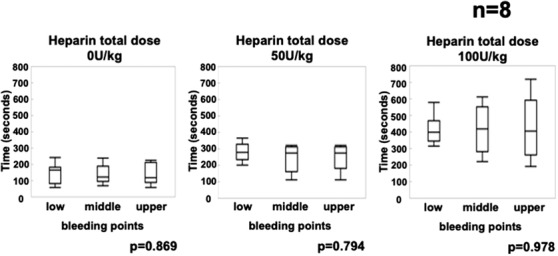
Changes in gastric mucosal bleeding time for each bleeding site

The median mucosal bleeding time was calculated as the median value of bleeding time in three bleeding sites, the upper, middle, and lower anterior walls of the gastric mucosa, for each swine. The median mucosal bleeding time was significantly increased by heparin administration (Figure 3: median mucosal bleeding time for no heparin vs. 50 U/kg vs. 100 U/kg: 152 vs. 283 vs. 423, respectively, *p* = 0.001). The median ACT and mucosal bleeding time at a heparin dose of 150 U/kg were not included in the results because only two pigs were administered heparin at this dose. In addition, there was no difference in the three bleeding points for each heparin administration (Figure 4).

## DISCUSSION

The practice using equipment such as virtual reality (VR) simulators have also been used in recent year since the development and surgical training with animals have problems because of issues with animal welfare. However, there are challenges in doing this because the equipment does not consider anatomical organs and does not use actual surgical instruments.^13^ On the other hand, animal models are still considered to be excellent teaching tools because they better reflect medical reality than devices.^14^ Additionally, conventional surgical training for practicing techniques on humans “Halstead training” has become more difficult to perform because of ethical reasons, and training with live animals is still common in our practice. However, inappropriate experiment models may require an unnecessarily large number of animals. The number of laboratory animals used in the experiments should be reduced, using adequate models optimized for each specific purpose.

Among commonly used laboratory animals, such as monkeys, dogs, and swine, the swine have become the most accepted surgical model because pigs have anatomical and physiological characteristics similar to those of humans and are larger than other animals.^15^, ^16^ However, swine have a problem that it is difficult to bleed during an endoscopic procedure.^5^, ^17^ Previous studies showed that platelets (×10^6^/ml) in humans were 265 ± 135 and platelets in swine were 350 ± 150,^7^ and there was a significantly higher function of coagulation and aggregation in swine than in humans.^6^, ^8^, ^9^ Piasecki and Wyatt showed that the area of the gastric mucosal surface supplied by a single mucosal artery is smaller in swine than in humans.^18^, ^19^ Also, it is possible that mucosa and submucosa of the digestive tract in swine may have fewer capillaries than in humans.^20^, ^21^ Therefore, we suggest that organ bleeding in swine may be difficult to induce.

ACT can indicate overall blood coagulability that is affected by activated partial thromboplastin time (APTT), prothrombin time, and platelets.^22^ Hence, there may be differences in the ACT values for each individual animal. Our study showed that ACT and mucosal bleeding were gradually increased by heparin administration and suggested that an intravenous bolus of heparin could be used to establish adequate animal models that bleed locally similarly to humans. In addition, we found that the amount of heparin could be adjusted while monitoring the ACT, and heparin could be safely used in large animals.

The main biological function of heparin is as an inhibitor of thrombin (FIIa and FXa) in the coagulation cascade, which allows the maintenance of blood flow, so heparin is used in various drugs.^11^ Heparin could be intravenously administrated, including bolus or persistence, and be subcutaneously administrated^11^, ^23^ in methods of heparin administration. A gastrointestinal endoscopy training in large animals may usually take approximately 30 min. Based on our results, local bleeding models by intravenous bolus administration with heparin may be effective for 20–30 min, we consider that the large animal model established in this study can be easily applied to endoscopic training.

Previous meta‐analysis study, the patients who underwent received heparin‐bridging therapy instead of anticoagulant had a significantly higher risk factor of post‐ endoscopic submucosal dissection (ESD) bleeding than the patients who did not receive it.^24^ However, to our knowledge, there was no study that showed the association between bleeding during ESD and heparinization in swine. On the other hand, Camus et al. reported a live animal bleeding model for training in endoscopic hemostasis, using anticoagulant for training in endoscopic hemostasis,^6^ and this model was created bleeding ulcer by endoscopic mucosal resection under pre‐administration clopidogrel or aspirin or unfractionated heparin. The therapeutic endoscopy such as ESD does not induce bleeding so often, but once the bleeding occurs, it has various levels of bleeding, from oozing to spurting, during dissection. This is because submucosa tissue and muscularis propria contain various sizes of venous perforators and arterioles. Therefore, we considered that standardized bleeding in therapeutic endoscopy is difficult to obtain. On the other hand, mucosal oozing can be obtained by simply traumatizing mucosa using biopsy forceps and its level is almost reproducible. The authors believe that standardized mucosal bleeding can be obtained with this technique.

There are several limitations to our study. First, it was not a clinical study on human subjects, but a preclinical animal study and only eight swine were used in this study. Second, we did not measure the APTT. Although the method of monitoring heparin generally utilized the measurement of the APTT,^25^ our study evaluated coagulation function in swine by measuring the ACT, which can indicate overall blood coagulability and is easy to measure than APTT. Third, the mucosal bleeding time was defined in the present study. The mucosal bleeding time was defined as the amount of time that elapsed before the bleeding stopped for 5 s after washing with 0.9% saline. However, it was unclear whether the mucosal bleeding time in our study was an accurate indicator of local bleeding. Fourth, we did not assess late bleeding, which was one of the complications that can occur after endoscopic surgery^26^; we need to evaluate late bleeding in further studies. Fifth, the efficacy of heparin additional administration or continuous intravenous was not verified. Given the short half‐life of heparin (45–60 min), it is necessary to evaluate whether that the optimal local bleeding models can continue by additional administration or continuous intravenous.

It is possible to perform experiments and surgical training that are more clinically relevant by using our animal models established in this study. Such local bleeding animal models may be used for various surgical and endoscopic procedures, simulation, training, and the development of medical devices.

## CONCLUSION

In conclusion, the present study suggests that swine local bleeding models may be successfully established by administering heparin intravenously. Furthermore, it is necessary to evaluate suitable for better surgical and endoscopic training using these established bleeding models. However, this animal model is expected to be widely used in various medical and life science fields for applications such as training for surgical endoscopic procedures and the development of medical devices and materials.

## CONFLICT OF INTEREST

The authors declare that they have no conflict of interest.

## ETHICS STATEMENT

This study was approved by the animal experiment committee of Osaka University (approval number: 02‐010‐000).

## FUNDING INFORMATION

None.

## Supporting information


**Supplemental Video 1A**: Gastric mucosal hemorrhage before heparin administration
**Supplemental Video 1B**: Gastric mucosal hemorrhage after heparin total 50 U/kg administration
**Supplemental Video 1C**: Gastric mucosal hemorrhage after heparin total 100 U/kg administration
